# Pressure transduction and fluid evacuation during conventional negative pressure wound therapy of the open abdomen and NPWT using a protective disc over the intestines

**DOI:** 10.1186/1471-2482-12-4

**Published:** 2012-03-24

**Authors:** Sandra Lindstedt, Malin Malmsjö, Johan Hansson, Joanna Hlebowicz, Richard Ingemansson

**Affiliations:** 1 Department of Cardiothoracic Surgery, Lund University Hospital, SE-221 85 Lund, Sweden; 2 Department of Ophthalmology, Lund University Hospital, Lund, Sweden; 3 Institution of Surgical Sciences, Faculty of Medicine, Uppsala University, Uppsala, Sweden; 4 Department of Medicine, Malmö University Hospital, Lund, Sweden

## Abstract

**Background:**

Negative pressure wound therapy (NPWT) has gained acceptance among surgeons, for the treatment of open abdomen, since very high closure rates have been reported with this method, compared to other kinds of wound management for the open abdomen. However, the method has occasionally been associated with increased development of fistulae. We have previously shown that NPWT induces ischemia in the underlying small intestines close to the vacuum source, and that a protective disc placed between the intestines and the vacuum source prevents the induction of ischemia. In this study we compare pressure transduction and fluid evacuation of the open abdomen with conventional NPWT and NPWT with a protective disc.

**Methods:**

Six pigs underwent midline incision and the application of conventional NPWT and NPWT with a protective disc between the intestines and the vacuum source. The pressure transduction was measured centrally beneath the dressing, and at the anterior abdominal wall, before and after the application of topical negative pressures of -50, -70 and -120 mmHg. The drainage of fluid from the abdomen was measured, with and without the protective disc.

**Results:**

Abdominal drainage was significantly better (p < 0. 001) using NPWT with the protective disc at -120 mmHg (439 ± 25 ml vs. 239 ± 31 ml), at -70 mmHg (341 ± 27 ml vs. 166 ± 9 ml) and at -50 mmHg (350 ± 50 ml vs. 151 ± 21 ml) than with conventional NPWT. The pressure transduction was more even at all pressure levels using NPWT with the protective disc than with conventional NPWT.

**Conclusions:**

The drainage of the open abdomen was significantly more effective when using NWPT with the protective disc than with conventional NWPT. This is believed to be due to the more even and effective pressure transduction in the open abdomen using a protective disc in combination with NPWT.

## Background

Treatment of open abdomen with negative pressure wound therapy (NPWT) in cases of abdominal sepsis and abdominal compartment syndrome results in a high rate of successful abdominal closure [1-5]. The primary goals of wound management include avoidance of mechanical contamination of abdominal viscera, active removal of exudates, estimation of third space fluid loss, and infection control [6]. NPWT involves application of topical negative pressure to the open wound. A non-adhesive perforated plastic barrier is placed over the viscera and extended laterally under the anterior abdominal wall. This first permeable layer is then covered with a polyurethane sponge and sealed with an airtight plastic sheet. An aspiration system is used to apply suction often ranging between 125 and 150 mmHg. The primary goal of this treatment is to remove contaminated fluid from the peritoneal cavity.

Temporary closure of the abdominal cavity with plastic bags, silicone sheets, absorbable and non-absorbable meshes sutured to the fascial or skin edges has not been found to facilitate permanent closure of the abdominal wall. Skin-only closure or split-thickness skin grafting may be used to cover the intestines and omentum [1,7,8]. The major drawback of these techniques is the formation of extensive ventral hernias requiring later treatment. The use of airtight dressings and NPWT to manage the open abdomen has improved care and the potential for subsequent closure of the open abdomen. However, the method has occasionally been associated with increased development of intestinal and enteroatmospheric fistulae [9-13].

We have previously shown that NPWT induces ischemia in the small intestinal wall [14]. We have also shown that placing a protective disc between the intestines and the vacuum source protects the intestines from ischemia [14]. Persistent ischemia in the intestinal wall could explain why conventional NWPT has been associated with development of fistulae. In the present study, we examine the differences in pressure transduction in the open abdomen and fluid evacuation with conventional NPWT and NPWT with a protective disc between the intestines and the vacuum source. To our knowledge, no such study has previously been conducted.

## Methods

### Experimental animals

Six domestic pigs of both genders, with a median weight of 60 kg, were used. The animals fasted overnight, but were given free access to water. The study design was approved by the ethical committee on animal experiments in Region Skane, Sweden. The study comply with the "Animal Research: Reporting In Vivo Experiments" ARRIVE guidelines.

### Anesthesia

All animals were premedicated with intramuscular ketamine (30 mg/kg) before being brought into the laboratory. Sodium thiopental (5 mg/kg), atropine (0.02 mg/kg), and pancuronium (0.5 mg/kg) were given intravenously immediately before surgery. A Portex endotracheal tube (7.5 mm internal diameter, Medcompare, South San Francisco, CA) was used for intubation. A servo-ventilator (Siemens Elema 300A, Stockholm, Sweden) was used for mechanical ventilation throughout the experiments. The ventilator settings used were: minute volume = 100 ml/kg, FiO_2 _= 0.5, breathing frequency = 16 breaths/minute, and positive end expiratory pressure = 5 cmH_2_O. Anesthesia and muscular paralysis were maintained by continuous intravenous infusion of 8-10 mg/kg/hour propofol (Diprivan, AstraZeneca, Sweden), 0.15 mg/kg/hour fentanyl (Leptanal, Lilly, France), and 0.6 mg/kg/hour pancuronium (Pavulon, Organon Teknika, Boxtel, the Netherlands).

### Data acquisition

Heart frequency and ventilator parameters were recorded throughout the experiments.

### Surgical procedure

A 30-cm midline incision was made on each pig. The V.A.C.^® ^Granu Foam™ abdominal dressing system (KCI, San Antonio, TX), was used. The visceral protective layer was cut to an appropriate size, extending into the paracolic gutters on both sides (about 30 cm wide and 35 cm long). A layer of polyurethane Granu Foam was placed on top of the visceral protective layer between the edges of the wound. The wound was covered with self-adhesive polyethylene drape, a track pad was inserted through the drape (both from V.A.C., KCI, San Antonio, TX), and then connected to a continuous vacuum source.

Pressure transduction was measured using a custom-made pressure gauge with saline-filled catheters. Pressure transduction probes were sutured to two intestinal ileal loops, one of which was sutured to the inner surface of the dressing, and the other at the anterior abdominal wall. Probe location was confirmed upon completion of the experiments.

A chest tube was inserted through the abdominal wall into the Pouch of Douglas, and 500 ml albumin solution was infused into the Pouch of Douglas to mimic the fluid in an open abdomen. NPWT was applied at pressures of -50, -70, and -120 mmHg with and without a protective disc between the intestines and the vacuum source. The amount of fluid evacuated into a canister was measured according to a scale. The abdomen was completely drained between each pressure setting before another 500 ml albumin solution was infused.

### The protective disc

The protective perforated plastic disc placed between the dressing and the intestines was soft, flexible, and approximately 3 mm thick.

### Calculations and statistics

Calculations and statistical analysis were performed using GraphPad 5.0 software (San Diego, CA, USA). Statistical analysis was performed using the Mann-Whitney test when comparing two groups, and the Kruskal-Wallis test with Dunn's test for multiple comparisons when comparing three groups or more. Significance was defined as, p < 0.05 (*), p < 0.01 (**), p < 0.001 (***), and p > 0.05 (not significant, n.s.).Given values are means and SEMs.

## Results

### Pressure transduction

**Figure 1:**
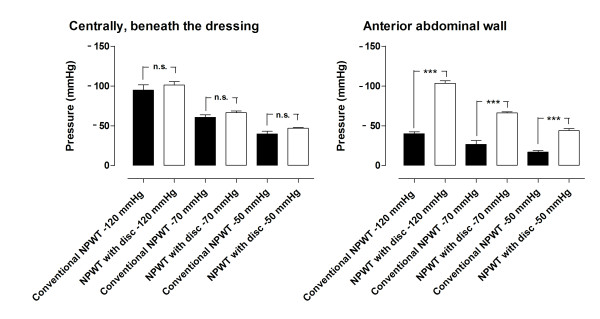
**Pressure transduction during NPWT**. Measurements were performed with conventional NPWT and NPWT with a protective disc between the intestines and the vacuum source. Negative pressures of -50, -70, and -120 mmHg were applied and the pressure transduction centrally, beneath the dressing and on the anterior abdominal wall was recorded. The results are shown as means ± the SEM of six experiments. Statistical analysis was performed using the Mann-Whitney test. Significance was defined as p < 0.05 (*), p < 0.01 (**), p < 0.001 (***) and p > 0.05 (not significant, n.s.).

Figure 1 shows the results of the pressure transduction measurements. At a pressure of -120 mmHg, the pressure centrally beneath the dressing was -95 ± 7 mmHg with conventional NPWT, and -101 ± 4 mmHg with the protective disc (n.s.). The pressure at the anterior abdominal wall was significantly higher with the protective disc than with conventional NPWT: -103 ± 3 mmHg vs. -40 ± 2 mmHg (p < 0. 001).

At -70 mmHg, the transduced pressure centrally beneath the dressing was -61 ± 3 mmHg with conventional NPWT, and -67 ± 2 mmHg using the protective disc (n.s.). The pressure at the anterior abdominal wall was significantly higher with the protective disc than with conventional NPWT: -66 ± 1 mmHg vs. -27 ± 4 mmHg (p < 0. 001).

At an applied pressure of -50 mmHg, the transduced pressure centrally beneath the dressing was -40 ± 3 mmHg with conventional NPWT, and -47 ± 1 mmHg using the protective disc (n.s.). At the anterior abdominal wall pressure was significantly higher with the protective disc than with conventional NPWT: -44 ± 2 mmHg vs. -17 ± 2 mmHg (p < 0. 001).

### Drainage

**Figure 2:**
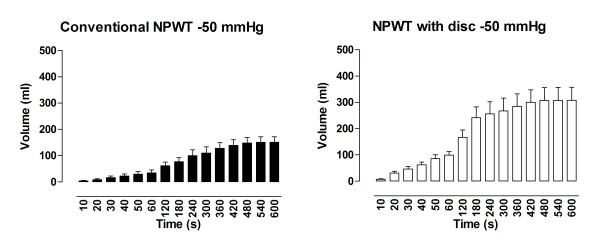
**Fluid removal measured over a period of 10 minutes during NPWT at-50 mmHg**. Measurements were performed with conventional NPWT and NPWT with a protective disc between the intestines and the vacuum source. The results are shown as means ± the SEM of six experiments. Statistical analysis was performed using the Mann-Whitney test. Significance was defined as p < 0.05 (*), p < 0.01 (**), p < 0.001 (***) and p > 0.05 (not significant, n.s.).

**Figure 3:**
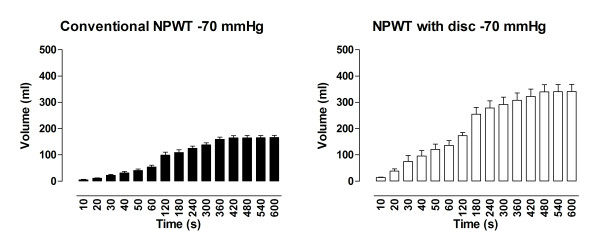
**Fluid removal measured over a period of 10 minutes during NPWT at-70 mmHg**. Measurements were performed with conventional NPWT and NPWT with a protective disc between the intestines and the vacuum source. The results are shown as means ± the SEM of six experiments. Statistical analysis was performed using the Mann-Whitney test. Significance was defined as p < 0.05 (*), p < 0.01 (**), p < 0.001 (***) and p > 0.05 (not significant, n.s.).

**Figure 4:**
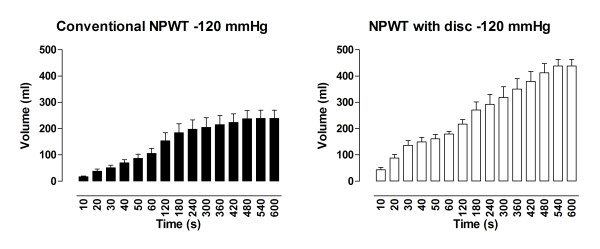
**Fluid removal measured over a period of 10 minutes during NPWT at-120 mmHg**. Measurements were performed with conventional NPWT and NPWT with a protective disc between the intestines and the vacuum source. The results are shown as means ± the SEM of six experiments. Statistical analysis was performed using the Mann-Whitney test. Significance was defined as p < 0.05 (*), p < 0.01 (**), p < 0.001 (***) and p > 0.05 (not significant, n.s.).

During application of -120 mmHg, the amount of fluid removed from the abdomen using NPWT with the protective disc was 439 ± 25 ml, compared with 239 ± 31 ml when using conventional NPWT (p < 0. 001) (Figure 2). At -70 mmHg, the amount of fluid drained from the abdomen using NPWT with the disc was 341 ± 27 ml compared with 166 ± 9 ml using conventional NPWT (p < 0. 001) (Figure 3). During application of -50 mmHg the amount of fluid removed from the abdomen using NPWT with the disc was 350 ± 50 ml versus 151 ± 21 ml using conventional NPWT (p < 0. 001) (Figure 4).

## Discussion

With the development of damage-control techniques and the understanding of abdominal compartment syndrome, the open abdomen has become more commonplace. Three scenarios commonly leading to an open abdomen are: peritonitis, expansion of the bowel during laparotomy, and increased intra-abdominal pressure in patients with severe abdominal compartment syndrome. Many trauma patients with intra-abdominal bleeding require damage-control surgery. This involves rapid assessment of the injuries and control of bleeding by direct suture/ligation, or gauze packing. The abdomen may be left open as part of the damage-control surgery, or bowel edema and/or gauze packing may simply preclude full fascial closure in these patients. The open abdomen requires temporary closure. If the abdomen is not closed in the early postoperative period, the combination of adhesions and fascial retraction frequently make primary fascial closure impossible, and a planned ventral hernia is often required. NPWT involves suction over a large polyurethane sponge under an occlusive dressing in the wound, which provides constant medial traction of the abdominal fascia. The technique also allows the abdominal wall to move freely toward the midline without interference from adhesions between bowels and the abdominal wall. NPWT also improves/facilitates drainage, which reduces the amount of peritoneal fluid and bacteria. Higher closure rates of the abdomen have been reported with NWPT than with other wound management techniques [6,15-18]. However, the method has occasionally been associated with increased development of intestinal fistulae and enteroatmospheric fistulae [9-13]. It has been suggested that the suction force of the vacuum induces an ischemic response in the underlying tissue that may promote development of fistulae.

There have been several reports over the years of excellent clinical results with NPWT [6,15,17,19-23]. However, in November 2009, the US Food and Drug Administration (FDA) issued a preliminary warning in view of reports of rare but serious complications associated with use of NPWT. In cardiac surgery, lethal complications following NPWT for postoperative deep sternal wound infection include right ventricle rupture and bypass graft rupture, with an incidence of 4 to 7% among patients treated for with NPWT deep sternal wound infection [21,24]. We have previously identified the cause of heart rupture in pigs using magnetic resonance imaging [21,24]. The heart was shown to be drawn up toward the thoracic wall, with the right ventricle bulging into the space between the sternal edges and the sharp edges of the sternum protruding into the anterior surface of the heart [24]. Placing multiple layers of paraffin gauze over the anterior portion of the heart did not prevent deformation of the heart. However, these events could be prevented by inserting a rigid plastic disc between the anterior part of the heart and the inside of the thoracic wall [24]. When using NPWT for treatment of the open abdomen, the mechanism may be similar, with herniation of the underlying tissue, i.e. bulging of the small intestines into the space between the wound edges, which might partially explain the induction of ischemia in the underlying intestinal wall during NPWT of the open abdomen [14]. Macroscopic changes in the small intestines lying close to the NPWT dressing in laparotomy wounds over 24 and 48 hours were recently studied in 70 kg pigs [25]. Half of the animals were treated with a protective thin plastic disc over the intestines, while the other halves were treated with conventional NPWT for open abdomen. Slight petechial bleeding was seen in the small intestinal loops lying close to the dressing in both groups [25]. The area of petechial bleeding was significantly larger after 24 hours, but especially after 48 hours, in the conventional NPWT group. In contrast, hardly any petechial bleeding was seen in the group treated with a protective disc over the intestines [25]. The area of petechial bleeding may indicate signs of ischemia.

We have previously shown that NPWT induces an increase in the blood flow of the peristernal soft tissue (i.e. skeletal muscular and subcutaneous tissue), and also that the change is related to local effects, since the blood flow 4.5 cm from the wound edge was not affected by the negative pressure [26]. The blood flow increased with increasing subatmospheric pressure in both subcutaneous and skeletal muscular tissue. When the area under the flow-distance curve was analyzed, covering a distance of 0.5 to 4.5 cm from the wound edge, a maximal net increase in the blood flow in muscular tissue was observed at pressures of -75 and -100 mmHg,[26]. A difference was observed in the profiles of the blood flow responses in the subcutaneous and the muscular tissue. The distance from the wound edge to the point at which the blood flow increased was shorter in muscular tissue than in subcutaneous tissue. This may indicate that pressure is transduced differently in soft, dense tissue, and that a less dense tissue collapses more easily when subjected to pressure. A zone of relative hypoperfusion was observed in the immediate proximity of the wound edge [26]. This zone was larger at high negative pressures, and was especially prominent in subcutaneous tissue. The size of the hypoperfused zone depended on the pressure applied, and expanded with increasing negative pressure. The changes in the peristernal wound blood flow caused by NPWT vary with the distance from the wound edge. A few centimeters away from the wound edge, the blood flow increased when subatmospheric pressure was applied. Conversely, in the immediate proximity of the wound, the negative pressure induced relative hypoperfusion [26]. These physiological events may also take place in the intestinal wall and in the omentum during exposure to negative pressures, leading to an ischemic zone in the intestinal wall that is in close contact with the NPWT dressing. This in turn could lead to the development of intestinal fistulae.

The primary goals of NPWT wound management include avoidance of mechanical contamination of the abdominal viscera, active removal of exudates, and estimation of third space fluid loss. The present study compared conventional NPWT with NPWT using a protective disc, placed between the intestines and the vacuum source. In previous studies we have shown that conventional NPWT induces ischemia in the small intestines close to the dressing, and close to the anterior abdominal wall. We have also shown that microvascular blood flow in the small intestines can be restored by placing a protective disc between the intestines and the vacuum source [14]. In the present study we show that NPWT with a protective disc drains the abdomen more effectively than conventional NPWT during exposure to negative pressures of -50, -70, and -120 mmHg. The most prominent difference was seen at -50 mmHg. It would be clinically advantageous to treat these patients at low negative pressures, where no ischemic response is seen, but good drainage of the abdomen is still achieved. A possible explanation of the differences between fluid evacuation with conventional NPWT and NPWT with a disc may be the more even pressure transduction at the anterior abdominal wall using NPWT with a disc compared with conventional NPWT, as shown in Figure 1. Pressure transduction did not differ between conventional NPWT and NPWT with a disc in the space between the wound edges directly beneath the dressing, but a difference was observed when comparing pressure transduction at the anterior abdominal wall, where more even pressure transduction was observed with NPWT with the disc, and essentially no pressure transduction was observed with conventional NPWT.

## Conclusions

Drainage of the open abdomen was significantly more effective using NWPT with a protective disc than using conventional NWPT, probably because the protective disc allows more even pressure transduction at the anterior abdominal wall. Abdominal drainage was significantly more efficient when a disc was used compared with conventional NPWT, especially at lower negative pressures (-50 mmHg). It may be clinically advantageous to treat patients with open abdomen at a low negative pressure, since high negative pressure has been shown to induce an ischemic response in the small intestinal walls close to the dressing.

## Conflicts of interests

The authors declare that they have no competing interests.

## Authors' contributions

SL, RI, JHle & MM carried out the experimental studies. SL drafted the manuscript. SL, JH, RI participated in the design of the study and performed the statistical analysis. All authors read and approved the final manuscript.

## Pre-publication history

The pre-publication history for this paper can be accessed here:

http://www.biomedcentral.com/1471-2482/12/4/prepub

## References

[B1] Schecter WP Ivatury RR Rotondo MF Hirshberg A Open abdomen after trauma and abdominal sepsis: a strategy for management J Am Coll Surg 2006 203 3 390 396 10.1016/j.jamcollsurg.2006.06.001 16931311

[B2] Swan MC Banwell PE The open abdomen: aetiology, classification and current management strategies J Wound Care 2005 14 1 7 11 15656456 10.12968/jowc.2005.14.1.26727

[B3] Deenichin GP Abdominal compartment syndrome Surg Today 2008 38 1 5 19 10.1007/s00595-007-3573-x 18085356

[B4] Barker DE Kaufman HJ Smith LA Ciraulo DL Richart CL Burns RP Vacuum pack technique of temporary abdominal closure: a 7-year experience with 112 patients J Trauma 2000 48 2 201 206 discussion 206-207 10.1097/00005373-200002000-00001 10697075

[B5] Brock WB Barker DE Burns RP Temporary closure of open abdominal wounds: the vacuum pack Am Surg 1995 61 1 30 35 7832378

[B6] Stevens P Vacuum-assisted closure of laparostomy wounds: a critical review of the literature Int Wound J 2009 6 4 259 266 10.1111/j.1742-481X.2009.00614.x 19719522 PMC7951520

[B7] Navsaria PH Bunting M Omoshoro-Jones J Nicol AJ Kahn D Temporary closure of open abdominal wounds by the modified sandwich-vacuum pack technique Br J Surg 2003 90 6 718 722 10.1002/bjs.4101 12808621

[B8] Schachtrupp A Fackeldey V Klinge U Hoer J Tittel A Toens C Schumpelick V Temporary closure of the abdominal wall (laparostomy) Hernia 2002 6 4 155 162 10.1007/s10029-002-0085-x 12424592

[B9] Bee TK Croce MA Magnotti LJ Zarzaur BL Maish GO Minard G Schroeppel TJ Fabian TC Temporary abdominal closure techniques: a prospective randomized trial comparing polyglactin 910 mesh and vacuum-assisted closure J Trauma 2008 65 2 337 342 discussion 342-334 10.1097/TA.0b013e31817fa451 18695468

[B10] Becker HP Willms A Schwab R Small bowel fistulas and the open abdomen Scand J Surg 2007 96 4 263 271 18265852 10.1177/145749690709600402

[B11] Fischer JE A cautionary note: the use of vacuum-assisted closure systems in the treatment of gastrointestinal cutaneous fistula may be associated with higher mortality from subsequent fistula development Am J Surg 2008 196 1 1 2 10.1016/j.amjsurg.2008.01.001 18355795

[B12] Trevelyan SL Carlson GL Is TNP in the open abdomen safe and effective? J Wound Care 2009 18 1 24 25 19131914 10.12968/jowc.2009.18.1.32139

[B13] Rao M Burke D Finan PJ Sagar PM The use of vacuum-assisted closure of abdominal wounds: a word of caution Colorectal Dis 2007 9 3 266 268 10.1111/j.1463-1318.2006.01154.x 17298627

[B14] Lindstedt S Malmsjo M Hansson J Hlebowicz J Ingemansson R Microvascular blood flow changes in the small intestinal wall during conventional negative pressure wound therapy and negative pressure wound therapy using a protective disc over the intestines in laparostomy Ann Surg 2012 255 1 171 175 10.1097/SLA.0b013e31823c9ffa 22104565

[B15] Cheatham ML Malbrain ML Kirkpatrick A Sugrue M Parr M De Waele J Balogh Z Leppaniemi A Olvera C Ivatury R D'Amours S Wendon J Hillman K Wilmer A Results from the International Conference of Experts on Intra-abdominal Hypertension and Abdominal Compartment Syndrome. II. Recommendations Intensive Care Med 2007 33 6 951 962 10.1007/s00134-007-0592-4 17377769

[B16] Malbrain ML De laet I, Cheatham M: Consensus conference definitions and recommendations on intra-abdominal hypertension (IAH) and the abdominal compartment syndrome (ACS)-the long road to the final publications, how did we get there? Acta Clin Belg Suppl 2007 1 44 59 24881700 10.1179/acb.2007.62.s1.007

[B17] Perez D Wildi S Demartines N Bramkamp M Koehler C Clavien PA Prospective evaluation of vacuum-assisted closure in abdominal compartment syndrome and severe abdominal sepsis J Am Coll Surg 2007 205 4 586 592 10.1016/j.jamcollsurg.2007.05.015 17903734

[B18] Svensson S Monsen C Kolbel T Acosta S Predictors for outcome after vacuum assisted closure therapy of peri-vascular surgical site infections in the groin Eur J Vasc Endovasc Surg 2008 36 1 84 89 10.1016/j.ejvs.2007.12.020 18294875

[B19] Amin AI Shaikh IA Topical negative pressure in managing severe peritonitis: a positive contribution? World J Gastroenterol 2009 15 27 3394 3397 10.3748/wjg.15.3394 19610140 PMC2712900

[B20] DeFranzo AJ Argenta L Vacuum-assisted closure for the treatment of abdominal wounds Clin Plast Surg 2006 33 2 213 224 10.1016/j.cps.2005.12.007 16638464

[B21] Petersson U Acosta S Bjorck M Vacuum-assisted wound closure and mesh-mediated fascial traction-a novel technique for late closure of the open abdomen World J Surg 2007 31 11 2133 2137 10.1007/s00268-007-9222-0 17879112

[B22] Shaikh IA Ballard-Wilson A Yalamarthi S Amin AI Use of topical negative pressure 'TNP' in assisted abdominal closure does not lead to high incidence of enteric fistulae Colorectal Dis 2010 12 9 931 934 10.1111/j.1463-1318.2009.01929.x 19438884

[B23] Ubbink DT Westerbos SJ Nelson EA Vermeulen H A systematic review of topical negative pressure therapy for acute and chronic wounds Br J Surg 2008 95 6 685 692 10.1002/bjs.6238 18446777

[B24] Malmsjo M Petzina R Ugander M Engblom H Torbrand C Mokhtari A Hetzer R Arheden H Ingemansson R Preventing heart injury during negative pressure wound therapy in cardiac surgery: assessment using real-time magnetic resonance imaging J Thorac Cardiovasc Surg 2009 138 3 712 717 10.1016/j.jtcvs.2008.11.068 19698860

[B25] Lindstedt S Malmsjo M Hansson J Hlebowicz J Ingemansson R Macroscopic changes during negative pressure wound therapy of the open abdomen using conventional negative pressure wound therapy and NPWT with a protective disc over the intestines BMC Surg 2011 11 10 10.1186/1471-2482-11-10 21529362 PMC3095529

[B26] Wackenfors A Gustafsson R Sjogren J Algotsson L Ingemansson R Malmsjo M Blood flow responses in the peristernal thoracic wall during vacuum-assisted closure therapy Ann Thorac Surg 2005 79 5 1724 1730 discussion 1730-1721 10.1016/j.athoracsur.2004.10.053 15854963

